# EU–Asian–American Partnership for a Third Industrial Revolution: Transitioning to High Productivity, Sustainable Infrastructures in the Age of COVID‐19

**DOI:** 10.1111/1758-5899.12918

**Published:** 2021-02-27

**Authors:** Vicente Lopez‐Ibor Mayor, Fazlun Khalid, Nafeez Mosaddeq Ahmed

**Affiliations:** ^1^ Former National Energy Commissioner for Spain; ^2^ Member of the Governing Council, United Nations Environment Programme; ^3^ Research Fellow, Schumacher Institute for Sustainable Systems

## Abstract

The COVID‐19 virus has caused a crisis for the world's economy and markets. The World Health Organization has declared the virus to be a global pandemic, meaning that it will have a sustained impact worldwide. In response to the shutdown of economies, governments across the world have implemented fiscal and monetary stimulus packages to counteract the disruption caused by the coronavirus. However, with many countries’ economies already slowing before the pandemic, the measures to combat the virus risk sending many countries into full scale recession for the first time since 2009, according to the European Commission. COVID‐19 has demonstrated the vulnerability of global supply chains and the need for more resilient infrastructures. Yet Europe cannot do this alone. The EU can only achieve this by strengthening ties and increasing trade cooperation with Asia and South America, in alignment with the values of sustainability. Prior to the COVID‐19 crisis, however, the EU has had major public disagreements around trade with both regions, essentially on environmental issues. By prioritising cooperation, the EU can work with developing countries in Asia and South America to take tangible steps towards environmentally sustainable production while boosting economic trade.

## Abstract 2

Strong EU–Asian–American partnerships in response to COVID‐19 can enable all sides to come out stronger and better equipped to tackle future environmental, health and economic crises.

The COVID‐19 virus has created an unprecedented crisis for the world's economy and markets. The virus has exposed a lack of trust in political institutions across the world and has raised questions as to whether they will be capable of delivering public goods and services. To maintain the social contract, political institutions must shift away from laissez‐faire ideology toward civic responsibility.

Estimates to date suggest that the period of social disruption could last as long as 18 months, if not longer – at least until a vaccine emerges. During this period, governments and economies will be increasingly strained under the pressure of needing to meet the needs of citizens amidst a prolonged slump of economic activity.

Indeed, with many countries’ economies already slowing before the pandemic, the measures to combat the virus risk sending many countries into full scale recession for the first time since 2009.

COVID‐19 has demonstrated the vulnerability of global supply chains and the need for more resilient infrastructures. It has also demonstrated the ever‐increasing ecological dangers of industrial expansion, which has amplified the risks of diseases jumping from insects or animals to humans. Without a change of course, COVID‐19 is a foretelling of a far more vulnerable and volatile future, one that would undermine the integrity of political institutions and accelerate widespread levels of distrust.

The key contribution of this paper will be to show that COVID‐19 has exposed the grave flaws in an international system which privileges profit over planet, resulting in competitive geopolitical dynamics that have undermined environmental goals by pitting rival economies against each other in a way that accelerates unsustainable forms of production. But there is an alternative that makes economic sense: by engaging in cooperation, the US and EU can work with developing countries in Asia and South America to take tangible steps towards environmentally sustainable production while boosting economic trade. It is clear that all sides need each other more than ever to help keep the strained global trading system alive. In so doing trust in political institutions can also be regained especially at a time when they are under increasing suspicion.

To develop this argument, we will first give an overview of how the COVID‐19 pandemic has demonstrated that prevailing laissez‐faire economic models are struggling to offer resilience to an age of complex global crises, indicating the need to redesign markets so that they work to meet the ‘public good’ goals of sustainability and longevity.

Importantly, the key claim is not simply that Europe must change but that such endeavors must be done in partnership with developing countries to avoid the mistakes of the current global system.

In order for the European Union to succeed in building the decentralised infrastructure for what Jeremy Rifkin has called a ‘Third Industrial Revolution’, it must engender new institutions for increasing collaboration and trade cooperation with emerging markets in Asia and South America, in alignment with the values of civic and ecological responsibility.

This will in turn require new approaches to sustainability for commodities like palm oil and beef, implicated in accelerating deforestation worldwide, which is today a major driver of vulnerability to disease outbreaks and pandemics. Under previous economic and political models, the call for large‐scale boycotts of problematic commodities has not only disrupted trade relations increasing economic vulnerabilities at a time of acute global crisis, but has undermined environmental goals.

Next, we review the three largest drivers of anthropogenic climate change: oil and gas production, agricultural production, and deforestation. On this basis, we will explore case studies of local nascent efforts to improve environmental protections in Malaysia and Brazil, to explore options for the way forward. These cases allow me to show that relying principally on voluntary standards to manage sustainability commitments is not as effective as standards enforced in partnership with state regulatory powers.

This suggests how the right mix of international and local policies can offer a way forward. We explore how COVID‐19 has enabled the creation of new public private‐partnerships, which could help balance economic needs with societal concerns about the environment, and ethical means of production.

This is followed by an overview of European responses to COVID‐19 in relation to energy policy. Beyond health responses, fiscal stimulus packages provide an opportunity for initiating a transformational and green recovery with the creation of green jobs and clean infrastructure.

The debate in Europe over pursuing a Green New Deals often focuses decarbonising national economies, without examining the issue of state regulatory infrastructure. However, it is imperative that Europe looks to transform the regulatory system itself to encourage decentralised power generation.

Finally, we will conclude by suggesting that this approach can pave the way for the establishment of strong EU–Asian–American partnerships. In the wake of the vast instabilities created by COVID‐19, this will enable all sides to come out stronger and better equipped to tackle future environmental, health and economic crises.

## Recovery processes and the Importance of human dignity

One of the most fundamental questions during the COVID‐19 pandemic is: how can we maintain human well‐being and dignity on a global scale amidst what is likely to be one of the worst economic crises in history? To successfully achieve this, we will need to reconsider the ‘normality’ of economics as we know it – if we do not, then there is a prospect of longer‐term civil unrest that could disrupt societies further.

The current approach to economic stimulus has been too constrained by traditional market economics. These approaches – which allow markets to do as they please – will need to be transcended. But this does not mean a lurch into extreme protectionism, which was already undermining the global economy prior to COVID‐19 and now endangers countries securing critical healthcare items. But it does require, in effect, the remaking and redesigning of markets so that while remaining as free and open as possible, they do so with a clear social purpose that can contribute to ‘public goods’ valued by citizens.

As part of this, the European Union will need to explore how to shape its economic recovery packages to ensure they not only address the emergency needs of the present moment, but help speed the transition to a more resilient economic future. This means recognising that the decentralised infrastructure for what Jeremy Rifkin has called a ‘Third Industrial Revolution’ can only be built through global partnerships with key emerging markets.

## Partnerships are necessary

The recognition that Europe cannot achieve its Green Deal ambitions alone is not a sign of weakness, but can open up new opportunities for collaboration. The Green Deal, ultimately, cannot be successful without the support of key partners in emerging markets. This requires strengthening ties and increasing trade cooperation with emerging markets in Asia and South America, in alignment with the values of civic and ecological responsibility (Weber, 2020).

Prior to the COVID‐19 crisis, however, the EU has had major public disagreements around trade with both regions, particularly on environmental issues. This has driven a breakdown of trust, undermining the possibility of joint mutually beneficial action.

This report proposes an EU–Asian–American sustainability partnership as the only viable economic response to COVID‐19. By working together in a coordinated and comprehensive manner to eliminate the biggest drivers of ecological crises – including the risk of pandemics – Europe, Asia and America can build new markets for public goods that can contribute to an inclusive economic transition which will create the foundations for a more resilient and sustainable future.

## Transcending the ‘normality’ of economics as we know it

The COVID‐19 pandemic has demonstrated that current market dynamics are not responding effectively to the complexity of current crisis. With global financial markets crashing down with accelerated speed, we must recalibrate markets to focus on sustainability and longevity. Yet ultimately any economic transition in this direction must do its best to alleviate the risks of another pandemic. We now know that deforestation, driven by industrial expansion, plays a key role in heightening the risk of disease outbreaks by increasing opportunities for animal interaction with human settlements.

Meanwhile, commodities like palm oil and beef have been harshly and rightly criticised for production techniques causing deforestation. This in turn has disrupted trade relations with major producers amidst calls for large‐scale boycotts. While the environmental concerns are valid, the solutions are questionable – as scientists have warned that boycotts of specific commodities are unlikely to displace rising demand, thus displacing that demand onto other problematic commodities. This results partly from extreme protectionist market dynamics in the context of a volatile financial system. It is my contention in this context that the only viable solution is a transformation of production.

In the age of COVID‐19, therefore, the way forward is for the EU to work with producers to build new more ethical markets, pioneering new sustainable production techniques, fostering positive free‐trade relationships that can create markets designed explicitly for the public good: to facilitate the exchange of mutually beneficial green goods and services as part of a global clean energy ecosystem.

In South America, initiatives in Brazil have shown how regenerative forms of farming and new regulations on deforestation can help curtail ecological damage. In Asia, major palm oil producer Malaysia has formally acknowledged the need to make improvements and redoubled its commitment to palm oil sustainability. With EU support, such initiatives show how both regions could continue to offer these critical commodities to global and European markets, helping to fuel sustainable consumption. In return, the EU can provide strong policy, financial incentives, and technological innovations integrating renewable energy, battery storage, Big Data, and social mobility to facilitate regional transitions.

As COVID‐19 lockdown conditions will continue for the foreseeable future, these new trade mechanisms may also pave the way for agreements on skills‐sharing and labour recruitment into key industries.

In these challenging times when the economic needs of the EU will be more urgent, it is important to pursue rebuilding trade connections without compromising on sustainability. The vision of a global Third Industrial Revolution will allow all three regions, the EU, South America and East Asia, to capitalise on new opportunities in developing markets focused on the most productive technologies of the future.

By remaking and redefining markets, this collaborative approach can rebuild trust in political institutions at a time when they will be under increasing suspicion.

## Current challenges

Before proposing the contours of this new tripartite EU–Asian–American partnership, we must understand the limitations of the current financial model, and the need for a global ‘Third Industrial Revolution’. Here we must look at the three main drivers of climate change and how there are linked to the existing structural problems that allowed the expansion of the coronavirus crisis far beyond any of us expected.

***Oil and gas***

It is well‐known that the oil and gas industry is the top driver of anthropogenic climate change and related environmental damage (Bach, 2019).

The role and responsibilities of oil and gas companies in climate change has become a rising concern and has provoked broad social backlash. Campaigns for divesting from fossil fuels, for instance, are proliferating worldwide; similarly, initiatives to coerce fossil fuel companies to keep their reserves underground are multiplying.

With specific regard to oil and gas companies, their contribution to global GHG emissions is substantial. The top 10 companies in terms of cumulative emissions all belong to the oil and gas industry. The biggest 60 oil and gas companies contributed to more than 40 per cent of global cumulative industrial emissions in the period 1988–2015; the top 10 accounted for almost 22 per cent, and the top 20 companies for more than 30 per cent (Grasso, 2019).

One large multinational corporation ExxonMobil helped to found and lead the Global Climate Coalition, an alliance of some of the world's largest companies seeking to halt government efforts to curb fossil fuel emissions. Exxon used the American Petroleum Institute, right‐wing think tanks, campaign contributions and its own lobbying to push a narrative that climate science was too uncertain to necessitate cuts in fossil fuel emissions. Such successful lobbying against climate policy and regulations, by ExxonMobil and others effectively halted the United States’ ratification of the Kyoto Protocol (Saleri et al., 2019).

### Agriculture

Industrial farming is the second largest driver of climate change after the oil and gas industry itself. Raising livestock for meat, eggs and milk generates 14.5 per cent of global greenhouse gas emissions, the second highest source of emissions and greater than all transportation combined. (Cameron and Cameron, 2017).

Most food crops eaten in the United States are produced on large land tracts planted in monocultures (single crops). Animals are raised separately from crops in large confinement facilities, eating specially formulated feeds made from grains and manufactured inputs, including antimicrobial drugs, rendered animal proteins, and arsenical compounds. Chemical fertilisers, irrigation, pesticides, herbicides, and new seed varieties were the immediate stimulants of the 20th‐century yield increases, but petroleum was, and continues to be, their essential energy source. Petroleum contributes most ingredients for manufacturing the pesticides and herbicides essential for controlling pests and weeds that thrive in monoculture production and supplies the energy to mine, process, and deliver phosphate and potash to farms. (Natural gas is the primary ingredient in most nitrogen fertilisers.) Petroleum also supplies energy to manufacture and operate the farm equipment that prepares the soil, sows and harvests crops, and irrigates fields. Finally, petroleum transports agricultural inputs such as pesticides and feed, agricultural products, and food. (Neff et al., 2011).

### Deforestation

Forests absorb roughly a quarter of the carbon dioxide emitted by human activity each year. Destroying all of the world's forests would release the same amount of stored carbon as burning all the planet's readily extractable fossil fuel deposits.

In 2018, the earth saw its fourth‐highest level of tropical tree loss since the early 2000s – about 30 million acres. The world’s forests contain more carbon than exploitable oil, gas, and coal deposits, hence avoiding forest carbon emissions is just as urgent as halting fossil fuel use.

Tropical deforestation is currently the third largest driver of climate change responsible for 10 per cent of global greenhouse gas emissions (UCS, 2012). There is also a direct responsibility on the part of Europe. Deforestation accounts for 15 per cent of the total carbon footprint of food consumption in EU countries (Pendrill et al., 2019). The authors suggest that this is indeed a substantial share which highlights the urgent need for consumption‐based accounts to include emissions from deforestation as well as implementation of policy measures that cross these international supply‐chains in order to effectively reduce deforestation emissions.

By the far the biggest global driver of carbon emissions induced by deforestation is beef production in Brazil, the rest of Latin America, and Africa, accounting for some 34 per cent of emissions. The next major driver, at around 20 per cent, is from oilseeds products such as vegetable oils. (Pendrill et al., 2019).

There is also a rather concerning pattern regarding the link between agriculture and climate change. The meat industry is heavily dependent on enormous quantities of soy which is used for animal feed to raise livestock. Shockingly, 75 per cent of the world’s soy is used for animal feed. With soy production expanding rapidly across South America, it is a global hotspot for deforestation led by large American companies such as Cargill and Bunge. These agribusinesses are creating industrial soy monocultures and by doing so they are endangering ancient native ecosystems and the wildlife habitat. (Yousefi et al., 2018).

Europe is the second largest market, after China for soy produced in South America (Yousefi et al., 2018). Deforestation is therefore a direct result of a long supply chain starting from South America and ending on people’s plates in Europe. While meat production requires large amounts of resources, the destruction of native ecosystems is not inevitable. There are over 650 million hectares of previously cleared land across Latin America. This land could be used for soy and cattle without destroying native ecosystems.

Out of the soy used for animal feed within the EU, 97 per cent is imported which puts the EU in a position in which it can enact significant change throughout the agricultural industry. Consequently, the EU has a great responsibility to make sure that the soy it buys is not contributing to the destruction of forests and native ecosystems.

In recent years, evidence has emerged that tropical forests are no longer a carbon sink but a carbon source. These rather shocking findings demonstrate the need for more drastic forest conservation efforts. The amount of carbon emissions from tropical regions are in fact bigger than the carbon removal that the region is able to achieve. This problem is particularly identifiable in Latin America, especially Brazil, a region responsible for the largest amount of emissions principally due to deforestation for cattle rearing (Baccini et al., 2017).

Stopping climate change is an incredible complex mission which requires us to reevaluate our dependence on technologies which are the main drivers of carbon emissions. We can no longer continue business as usual since the dynamics of deforestation are increasingly inseparable from the growing demand for food from consumers in the most developed countries.

Standing forests pull moisture out of the ground and release water vapor to the atmosphere, regulating local, regional and global precipitation patterns and acting as a natural air conditioner (Gustin, 2019). In contrast, cutting down tropical forests increases local surface temperatures by up to 3°C. These ‘climate regulation’ effects of tropical forests make their conservation essential to protect food and water security.

If we continue on our current course, the risks are grave. However, in the light of recent research findings it is also crystal clear that putting an end to the rampant destruction of the forests on which planetary ecosystems depend is completely avoidable.

Recent research suggests that, in order to have a chance of limiting warming to 1.5°C, we cannot emit more than about 750 billion tons of CO2 in the coming century (Gustin, 2019). The carbon in readily exploitable fossil reserves could release 2.7 trillion tons of CO2 up to 2100. By comparison, forests store enough carbon to release over 3 trillion tons of CO2 if destroyed. And climate change itself makes forests more vulnerable, including to uncontrollable wildfires.

Given the relative ineffectiveness of previous approaches on deforestation, to tackle the above‐mentioned challenges, Europe should consider fostering new opportunities for cooperation with producer countries to tackle such trends in deforestation. We will explore how the case studies of Malaysia and Brazil suggest potential pathways for such partnerships.

## Vision of a global ‘Third Industrial Revolution’ in the age of COVID‐19

### Case studies of Malaysia and Brazil

- Malaysia leading the way in East Asia with its commitment to sustainable palm oil

Today the majority of the world’s palm oil – 85 per cent – originates from Malaysia and Indonesia (Rosner, 2018). Malaysia has been cultivating palm oil for more than a century. Originating from West Africa, oil palms were introduced into Indonesia in 1848 and Malaysia in 1875 under Dutch and British colonial rule. The first commercial oil palm estate was established in Selangor in 1917 by a French plantation owner, Henri Fauconnier (Tang and Al Qahtani, 2019). Although oil palm plantations slowly expanded following independence, it was only during the 1960s that oil palm plantation really accelerated. Palm oil was actively promoted by the agricultural diversification programme of the Malaysian government.

The income from palm oil enabled Malaysia to develop its infrastructure, pave roads, improve telecommunications and build schools and hospitals. Rural employment increased and poverty declined.

While oil palm cultivation in Malaysia continued to expand from 1973 to 2010, it is important to recognise that deforestation did begin to slow down from the mid‐1980s. Scientists are unsure as to why this happened, with some speculation that it was because oil palm planting shifted away from newly cleared forests, to land that had been previously used for other agricultural commodities (e.g., rubber, coconut, cocoa) when they became less profitable than palm oil. Others note that economic diversification and poverty reduction also allowed other industries to flourish (Miyamoto et al., 2014).

With oil palm expansion, there have has been rising concern about how plantations are being established on land taken from tropical forests that housed endangered species such as orangutans. However, there have emerged a number of initiatives to attempt to conserve forests and transition to sustainable forms of palm oil production.

Let us examine two of the certification bodies relevant to the issue of sustainability, the Roundtable on Sustainable Palm Oil (RSPO) and Malaysian Sustainable Palm Oil (MSPO), both of which we shall examine in more detail.

### The Roundtable on Sustainable Palm Oil (RSPO)

The Roundtable on Sustainable Palm Oil was established in 2004 as a voluntary corporate‐led initiative with the objective of promoting the growth and use of sustainable oil palm products through global standards and proper oversight mechanisms. The standard was established in 2004 following reports that oil palm plantations were causing rainforest destruction and land grabs, where legal protections for the environment and indigenous communities were weak and enforcement of the law was marginal.

The Roundtable on Sustainable Palm Oil (RSPO) became one of the most recognised certification schemes for palm Oil.

However, the RSPO has been criticised for failing to provide sufficiently robust regulation. One study has found that RSPO‐certified plantations perform no better than non‐RSPO estates on a series of sustainability metrics, including species and habitat conservation, as well as social benefits to local communities (Nathan, 2017).

Other issues with RSPO include its focus on larger plantations as they have not been very effective in reaching out to the smallholders. Another problem is the RSPO’s low area of coverage: only one‐fifth of palm oil is certified by it. Many growers were discouraged by the cost of complying. Another of the sustainability measures where the RSPO is lacking is in helping conserve biodiversity because its plantations have been accused of pushing out the critically endangered Bornean orangutan.

One of the most significant weaknesses of RSPO is that it remains a voluntary verification system, and therefore non‐compliance does not necessarily trigger strong enough sanctions to incentivise corrective behaviour. This has led to accusations that its standards are too lenient and that it has little power to enforce them.

Most importantly, it is acceptable within the RSPO standards for plantation companies to clear peatlands which have are carbon rich and so‐called secondary forests – even if those forests are full of wildlife. Such flaws mean that some RSPO certified plantations have been associated with deforestation, climate emissions from clearance on peatlands and human and labour rights violations, which leaves the credibility of RSPO certification in question. (Morgans et al., 2018).

While voluntary standards such as the RSPO do address existing operations, studies have shown that it is beneficial to have a mix of both voluntary and mandatory regulation to ensure the sustainable management of both present and future plantation practices (Mukherjee and Sovacool 2014).

### Malaysian Sustainable Palm Oil (MSPO) certification

In January 2015, Malaysia established its own certification body to tackle the issue of sustainability, Malaysian Sustainable Palm Oil (MSPO), established for the management of palm oil plantations, smallholdings and palm oil processing facilities in Malaysia. The programme aims at ensuring the sustainability of palm oil estates which are 100 acres or more in size. When first established, it was a voluntary scheme, similar to more well‐known international schemes such as the Roundtable on Sustainable Palm Oil (RSPO), which is also voluntary and targeted largely at major corporate producers. The MSPO certification approach was more explicitly designed to be accessible to smallholder farmers, who are often excluded from the RSPO due to cost and bureaucratic problems (Morgans et al., 2018).

In September 2018, with the arrival of a new administration, MSPO was for the first time declared a mandatory, government‐backed national scheme aimed at providing the new government the power to enforce sustainable palm oil production, conserve forests and preserve key wildlife habitats. However, there has been a clear diplomatic and communications gap between Malaysia and Europe. The European Union continued to speed ahead with its legislation on biofuels which, six months later, culminated in a de facto ban on palm oil for biodiesel.

Since then, Malaysia has scaled up the MSPO scheme, managing to certify about 42 per cent of the country’s palm oil areas by August 2019, and aiming to achieve 70 per cent certification by earlier this year. So far, there has been little international interest in assessing or evaluating MSPO. However, independent conservationists who have visited ‘best of category’ cases of the MSPO scheme have confirmed their positive initial impressions of success in terms of sustainability, forest conservation and labour rights for migrant workers (Hii, 2017).

Despite these certification schemes we have seen the environmental concern about deforestation lead the European Union to ban the use of palm oil to make biofuels by 2020. The EU ban on palm oil favours alternative crops like rapeseed and soybeans that are grown in Europe as a source of oil for biofuel (Keating, 2019).

A major obstacle here is that as MSPO is a national certification scheme, there have been little to no points of contact or verification between the EU and Malaysia which would permit a process of independent monitoring that could satisfy international observers as to the reliability of the MSPO standard. This has been reinforced by recent EU efforts to consider strengthening its opposition to palm oil and other commodities implicated in contributing to deforestation.

Yet the EU policy in its current form is in tension with the scientific literature raises some questions as to whether solely limiting imports in tackling deforestation can be truly effective. A landmark report by the International Union for Conservation of Nature (IUCN) found that banning palm oil would likely cause more harm to the environment only displacing the global biodiversity losses instead of stopping them (Meijaard et al., 2018). This is because a palm oil ban would increase the production of other oil crops, such as rapeseed, soy or sunflower, which require up to nine times as much land to produce than palm oil, in order to meet the global demand (Pendrill et al., 2019). These crops store less CO2 than palm oil, require more fertiliser and pesticides and have lower productivity and shorter lifespan compared to oil palms (Fassler, 2016).

Director General of the IUCN, Inger Andersen has therefore called for urgent and ‘concerted action to make palm oil production more sustainable, ensuring that all parties – governments, producers and the supply chain – honour their sustainability commitments’ (IUCN 2018).

A major study in *Annual Review of Resource Economics* has provided a definitive analysis of the challenges, corroborating these findings. The *Annual Reviews* study is worth noting as it is one of the most authoritative analyses of the best scientific literature to date. It confirms that the key challenge is related to the efficiency of palm oil, relative to land, water, energy and fertiliser inputs: ‘The global demand for vegetable oil will continue to grow. Against this background, banning or curbing oil palm cultivation is not a realistic option. Given oil palm’s high land productivity, meeting the rising demand only through other oil crops would entail even more land‐use change and natural habitat loss’ (Qaim et al., 2020, p. 322). This analysis confirms the IUCN’s analysis that an approach focused only on limiting palm oil imports could actually drive greater rates of deforestation overall (Qaim et al., 2020).

A similar conclusion was reached by a team of University of Bath scientists, who specifically examined the potential impact of a palm oil ban, and whether alternatives could offer an environmentally viable replacement to meet demand. They found that this would increase the production of other oil crops, such as rapeseed, soy or sunflower, which require up to nine times as much land to produce than palm oil, in order to meet the global demand. These crops store less CO2 than palm oil, require more fertiliser and pesticides and have lower productivity and shorter lifespan compared to oil palms. The team, publishing their findings in *Nature*, conclude that in the near to mid‐term, policy should be directed at ensuring the sustainability of production because import restrictions would be ineffective in stopping deforestation or protecting the environment (Parsons et al., 2020).

There is another important side‐effect of the EU’s current approach which has played the biggest role in fostering distrust. The *Annual Reviews* paper finds that some 50 per cent of the worldwide oil palm land is managed by smallholders, and that focusing purely on import restrictions can end up penalising some of the most vulnerable households in developing countries. The palm oil industry, the study notes, has played a key role in increasing incomes, generating employment, and reducing poverty among local communities across these countries: ‘Especially in Southeast Asia, oil palm has contributed considerably to rural income growth and reduced poverty among farmers and workers’ Qaim et al., 2020, p. 337). Therefore, there is a risk that an approach premised simply on reducing imports of palm oil could have a detrimental impact on the UN Sustainable Development Goals (SDG), endangering the livelihoods of hundreds of thousands of smallholder farmers and the local communities they are embedded in (Qaim et al., 2020).

Rather than bans, avoiding further palm oil related deforestation will deliver more gains for biodiversity overall (Nazmi, 2020). So, the solution going forward is for the EU to find ways to work with Malaysia to help learn from best practices and take advantage of technology and experience Europe has to offer. This partnership may mean giving Malaysia more time to implement an agreed series of measures for producing sustainable palm oil, which both parties agree upon.

- Transforming cattle rearing methods in South America with Brazil setting an example

For South America, deforestation is possibly the most crucial environmental concern threatening the region. A study published by the journal of the Society for Conservation Biology found that the rise of global food demand and South America’s position as a key provider have aided the expansion of commodity production into forests (Gasparri and Waroux, 2014). This is not only impacting the Amazon but also dry forests and woodlands across the region affecting countries such as Bolivia, Paraguay and Argentina.

This partnership could include working with Brazil to create more sustainable cattle rearing methods. For the survival of the Amazon, improving Brazil’s environmental protection schemes is vital.

An article published by the *Nature* research journal argues that the international community also bears a responsibility when it comes to protecting the Amazon and must do more to encourage the government of Brazil to improve environmental protections. (Fuchs et al., 2019). The authors suggest that initiatives such as REDD+, provide mechanisms for coordinated action by rewarding developers and other actors financially for not clearing forests. Regulatory interventions trying to limit environmental degradation and its extent, are also needed even though they are often controversial and dependant on political circumstances.

The study provides Brazil’s Soy Moratorium, which received a lot of criticism despite its positive environmental impacts, as an example of these interventions (Reuters, 2019). Soy Moratorium in which major traders agreed not to buy soya grown on lands deforested after July 2006, managed to reduce the conversion of forest to cropland. What made the efforts successful was the various monitoring systems deployed to enforce it including police helicopters to track illegal logging. The authors warn that under changed political leadership due to Bolsonaro’s presidency, these achievements are under a threat given the rise in rogue logging.

Another example of the conservation efforts in Brazil is the Cerrado Manifesto (2017), several groups including Greenpeace, the World Wildlife Foundation and Brazilian research group IPAM called big multinationals to preserve the biome. Currently, the Cerrado Manifesto is endorsed by almost 140 international companies and institutional investors. It calls investors and companies to send a clear market signal of the importance of widespread industry support to end the deforestation in the Cerrado, Brazil’s vast tropical savanna ecoregion. The manifesto urges companies to adopt sustainable land management practices and mitigate financial risks related to climate change.

The companies that have signed the manifesto include big names such as McDonalds, Unilever, Nestlé and L'Oréal and have agreed to support measures that eliminate native vegetation loss from their supply chains in Cerrado (Spring, 2018). However, in comparison to the Soy Moratorium, the manifesto did not require signatories to stop buying agricultural products from newly deforested areas.

A study published by the World Development Journal explores the private agreements initiated in 2006 and 2009 to limit the purchase of soy and cattle linked with deforestation in the Brazilian Amazon (Le Polain de Waroux et al., 2017). The authors examined whether such policies generated negative spillovers including leakages of agricultural activities and deforestation to less‐regulated areas. What they found was that as beef importers moved away from exporters with the most rigorous deforestation regulations, supply from these regions was taken up by domestic markets. Thus, the redistribution of beef consumption and trade patterns can create deforestation loopholes which must be acknowledged.

The results demonstrate the complexity of production and trade responses to changing environmental regulations, and the need for more sophisticated modelling to understand the global impacts of local regulation changes. From an environmental policy perspective, this means minimising potential loopholes in both private and public regulations that accommodate continuing deforestation. Overall, this will require greater public and private co‐operation and international environmental governance efforts to synchronise regulations across all commodities, actors and regions, the authors conclude.

An article published in the Environment and Society journal suggested that over the past decade Brazil has managed to reduce its deforestation rates significantly while increasing its agricultural production, mostly soy and cattle (Zycherman, 2016). The authors argue that although this is partly due to global economic trends and environmental policies, new cultural values among the producers must also be counted towards the success. In the wake of more strict deforestation regulations and emerging agricultural opportunities, new relationships have been formed between farmers and ranchers changing their attitudes towards the land, and new technological advancements have also contributed positively towards this shift.

A study published by the Global Environmental Change journal argues that countries with a sizeable stock of grazelands, including Brazil, Argentina and Mexico, could increase land yields while remaining largely specialised in pasture‐based systems (Vale et al., 2019). In contrast to intensive beef farming, in traditional pasture‐based systems cattle are raised freely, often with access to running water and shading. This would call for a middle‐ground intensification approach such as the pasture‐based intensification currently pursued in Brazil. This alternative approach would result in both environmental and economic gains, particularly in the light of the demand for beef steadily shifting toward niche markets.

## New public and private partnerships emerging during the COVID‐19 crisis

The analyses of developments in Malaysia and Brazil, as well as the available scientific literature on the impact of boycotts, suggest that there is an important gap to be urgently filled in order to put an end to deforestation. While there is a strong evidence‐base suggesting that a ban on palm oil would be highly counterproductive for deforestation, the implications of this analysis has relevance for how other problematic commodities are viewed.

Overall, policy approaches premised exclusively on the goal of preventing specific commodities from entering markets would appear to be an ineffective solution because it fails to address the underlying drivers of demand, and does not account for that demand to continue growing by switching across different commodities.

This analysis thus highlights the inherent pitfalls of a purely market‐focused approach which relies principally on the private sector to take appropriate sustainability action, which is also a major limitation of the RSPO framework on palm oil. For instance, an article published in *Nature Climate Change* reviews some of the recent private sector commitments that aim to eliminate deforestation from a company’s operations or supply chain, only to conclude that most of these fall short on several fronts (Lambin et al., 2018). In order to mitigate climate change and biodiversity loss, a significant reduction in global deforestation is crucially needed. However, this requires greater transparency and commitment on the behalf of the private sector. This in turn can only be achieved against the backdrop of sufficiently robust government‐backed enforcement capabilities – in other words, we need a far stronger legal and regulatory framework provided by national governments.

The COVID‐19 crisis has already demonstrated how we must explore new avenues of public and private partnerships in order to tackle the challenges posed by climate change. In the aftermath of the pandemic, this is possibly more crucial than ever before. In the case of tackling deforestation, a major driver of the risk of disease outbreaks, the MSPO model in Malaysia hints at the kind of international partnerships that could be most fruitful: local sustainability and conservation certification schemes, supported by legally enforceable mandatory regulation, developed in partnership with international allies such as the EU who can provide financial support and scientific expertise.

This suggests that the most effective point of action, which the EU has so far neglected, is encouraging new partnerships in Asia and Latin America which are explicitly designed to facilitate the emergence of scientifically validated local policy regimes that protect and advance sustainable production processes.

An extensive modelling study on the challenge facing the palm oil industry in the *Proceedings of the National Academy of Sciences* underscores the need for such joined‐up thinking. Although focused on palm oil, these findings have important broad implications across industries implicated in deforestation. Finding that ‘simply limiting palm oil production or consumption is unlikely to halt deforestation’ in Malaysia and Indonesia, the study concluded that ‘in the absence of active forest conservation incentives … Targeting just a single driver of deforestation … opens room for other drivers of deforestation to operate more actively in the absence of a forest protection plan’ (Taheripour et al.. 2019, p. 19198). Its core implication is that the most powerful approach to stopping deforestation is not in targeting any particular commodity, but in *incentivising forest conservation efforts*.

This crucial scientific finding fits well with the recommendations of the *Annual Reviews* study, which calls on policy makers to develop ‘efficient legal and institutional frameworks in oil palm‐producing countries’ (Qaim et al., 2020, p. 335). Their analysis demonstrates that the EU’s approach suffers from a major gap from an environmental risk perspective: that of ensuring sustainability at source.

In this context, the European Union should consider reviewing its current approach and adapting it toward one focused more on working with producer countries to develop regulatory frameworks and incentives that would both limit imports of unsustainable palm oil, while simultaneously supporting and encouraging sustainable production.

These frameworks should encompass a number of areas: improving yield productivity using new sustainable production techniques; doing so within the clear delineation of protected forest lands combined with strong rules on use rights, prohibitions, and effective sanction mechanisms; recognition of customary land rights of local communities; robust sustainability certification along with verifiable monitoring mechanisms; and successful inclusion of smallholder farmers. The most important condition for these frameworks to be effective is that they cannot simply be imposed from outside with a ‘one‐size‐fits‐all’ approach. The *Annual Reviews* authors urge that: ‘The appropriate policy mix needs to be adjusted to the local context” (Qaim et al., 2020, p. 337).

With the right approach, the study suggests, successful policy mixes developed in Southeast Asia could also provide important learnings for production of commodities outside the region, whether it be palm oil, other vegetable oils or beef: ‘This is relevant for Southeast Asia, but also for Africa and Latin America, where much of the future oil palm expansion is expected to occur’ (Qaim et al., 2020, p. 335).

Speaking directly to this issue, a study published by the Global Environmental Change looks at the rise of public and private zero‐deforestation commitments in the context of South America and how they are opening a new collaborative space in global forest governance (Furumo and Lambin, 2020). Governments around the world are aiming to reduce national greenhouse gas emissions by partnering with companies committed to eliminate deforestation from supply chains to protect and restore forests.

The proliferation of zero‐deforestation initiatives has created new opportunities for policy synergies but also led to increasingly complex regulatory landscape. Using post‐conflict Colombia as a case study, the authors found that governments can indeed provide significant directionality among the proliferation of zero‐deforestation initiatives. Consequently, public‐private partnerships allow governments to amplify their efforts by aligning transnational activities with national priorities.

## New economic approaches

In return for EU financial and scientific support for new sustainable production practices across Asia and Latin America, the opportunity for new international trade relationships can emerge. This could provide the EU favourable access to the world’s most exciting emerging markets, and also generate new opportunities to mobilise the Third Industrial Revolution not just within the EU’s borders, but within Southeast Asia and Latin America.

These two regions are home to some of the world’s fastest growing economies. Resolving the diplomatic hurdles posed by the disagreements about sustainability would finally allow the EU to play a greater role in these regions, thereby positioning the bloc to benefit from the most economically dynamic countries. In the context of COVID‐19, this could open up important new avenues for economic revitalisation.

The EU’s Third Industrial Revolution agenda can provide an important framework for ensuring that this process unfolds in a way that advances cutting‐edge technologies while adhering to stringent sustainability goals.

The COVID‐19 pandemic has caused an unprecedented crisis for the world's economy and markets. The virus has exposed a lack of trust in political institutions across the world, and raises questions as to whether they will be capable of delivering public goods and services. To maintain the social contract, political institutions must shift away from laissez‐faire ideology toward civic responsibility.

If the world is to address the profound challenges and problems which confront us today, ‘business as usual’ is not an option. COVID 19 has shown that radical solutions are necessary and we must make radical efforts to find them.

When the 2009 financial crisis hit, it shook the public’s faith in the world’s economic system, and also confidence that policy makers knew how to manage it. Since that time policy makers have adopted some new approaches and economic analysis has become more nuanced.

COVID 19 has shown that these processes have not yet gone far enough because policy makers are still operating with an economic model and forms of policy that are from the past. More radical rethinking is required (McKibbin and Fernando 2020).

It is necessary for European economic policy to balance economic needs with societal concerns about the environment and ethical means of production. European societies have increasingly opposed the mercantilist advances of corporate culture. There is a demand that economic decisions and economic policy must be under the control of societal forces and policy debates should be hashed out in social institutions and through political processes.

As rapid technological change is transforming economies and innovation from consumer goods to advanced electronics, there is also concern about the dominance of multinational companies. Their market dominance, which is unprecedented in recent times, raises questions about their role in society. There is a rising debate in many countries about the impact of new technologies on issues ranging from democracy to mental health. New patterns of globalisation are also emerging.

As large multinational corporations dominate global production and supply chains there is also acknowledgement that investment and trade continue to shift to the south and east of the world. Most advanced economies have higher levels of private debt than in the past, higher returns to holders of share capital, and in some cases larger financial sectors relative to the rest of the economy.

The 2008 financial crisis exposed serious flaws not just in financial regulation but in the credit‐based form of growth which preceded it. Its effects continue to play out. For most countries, the recovery after the recession was among the slowest on record. Economic growth has been restored in the last few years, but it remains generally fragile, still dependent on the low interest rates and expanded central bank balance sheets.

Productivity growth has stalled in some countries, and is historically low in many others; innovation at the technological frontier is no longer being diffused to the rest of the economy as it was in the past.

There has also been a rise on inequality in the past several decades, particularly between the incomes of the top 1 per cent of the population and those of the rest of society. A surge in wealth inequality, in particular, has grown, in large part due to the appreciation in the value of assets, itself a cause of financial volatility.

In many countries, unemployment remains high, particularly for young people. Most developed economies have seen an increase in under‐employment and insecure and precarious work of different kinds, from self‐employment and part‐time work to very short term contracts.

Although economic policy is the prerogative of national governments, there is also an important international element as well. In a globalised economy of complex supply chains and trading relationships, production and consumption patterns in one country powerfully impact on others, and many economic outcomes cannot be determined solely through national action. So, there is a vital need to achieve new international agreements and coordination mechanisms in areas such as environmental degradation, labour standards and tax policy which can ensure that economic goals in one country are not met at the expense of others, and national policy is enhanced by international cooperation.

It is no longer acceptable for economic progress to be solely viewed as based on individual, material prosperity. Now it must include social wellbeing, community wellness and environmental protection. This will require doubling down on the EU’s Green Deal domestic commitments, while simultaneously reframing its conceptualisation of the Third Industrial Revolution as a unique EU product with huge foreign export potential. Rather than keeping the Revolution to itself, the EU can not only spearhead implementation within the Union, but export it to emerging markets like Southeast Asia and Latin America.

## Energy challenges for green recovery

The 2018 report of the United Nations Intergovernmental Panel on Climate Change (IPCC) declared that global emissions of greenhouse gases must be halved by 2030, in order to achieve the international goal of keeping the average surface temperature rise to 1.5 degrees celsius, and reach net zero by around the middle of the century (IPCC 2018).

But this environmental crisis is made even more challenging because of the need to tackle simultaneously a series of other global environmental problems, including biodiversity loss, soil degradation, and air and marine pollution.

Globally, there are problems because there is far too much fossil fuels in the global energy system. It should be acknowledged that there is a need for ‘just transition’ and the transition away from fossil fuels needs to happen at an accelerated pace. At the moment research indicates it is not happening fast enough (Figueres et al., 2017).

In order to achieve a transformational and green recovery, it will be necessary to take action on a longer‐term agenda to address climate change, avoid habitat loss and fragmentation, reverse the loss of biodiversity, reduce pollution and improve waste management and infrastructure.

The EU is adopting policy of major economic support programmes to cope with a health crisis that has caused a global social and economic shock. Beyond direct health responses, fiscal stimulus packages provide an opportunity for initiating a transformational and green recovery with the creation of green jobs.

Over the past several decades the global energy sector has experienced a dramatic transformation. There are major improvements in efficiency and the introduction of renewables. Rifkin’s Third Industrial Revolution calls for the integration of the decentralised renewable energy transition with the information revolution and Big Data, to facilitate the dawn of a new era of industrial productivity centred around the empowerment of local households, communities, and businesses. Yet Rifkin’s work does not address the urgency of robust regulatory frameworks to facilitate the rapid spread of such new integrated infrastructures.

The debate in Europe over pursuing green new deals often focuses on decarbonising national economies, without examining the issue of state infrastructure. However, it is imperative that Europe look to transform the power system itself to encourage decentralised power generation.

For the past several decades, power generation has been the domain of centralised power plants which provide the majority of electricity needs. However, this centralisation has led to many insufficiencies in the power grid: a large amount of energy is lost during transmission, reliability is not always guaranteed and traditional fuels like coal, nuclear power and gas have fallen into disrepute due to their negative impact on climate.

Conventional centralised electricity generation lacks variability to adjust to challenges posed by recent changes in our modes of energy consumption.

For much of the past century majority of electricity production has been taking place in remote locations, with distribution networks established to supply urban centres and residential communities. But due to the procedures involved and the distances travelled, there is a great deal of wasted energy. Even at the consumer end there is inefficient electricity use. (Hageman 2020).

Grid maintenance and construction are expensive and are prone to power failures. In Germany, 40 per cent of the generated electricity is typically produced from coal, one of the most polluting resources on the market. There is also a huge loss with more than 50 per cent of the energy simply vented in the form of heat. (Kunzig, 2015) In addition, fossil fuels are a scarce resource. Over the long term relying on coal or oil is not sustainable due to high environmental costs. Therefore, looking ahead the traditional model of centralised electricity generation and distribution must be re‐evaluated.

There are new more sustainable and efficient forms of energy distribution that produce electricity where it is consumed. While large grids produce failures and inefficiencies, decentralised energy and smaller grids appear to be a more reliable and cheaper alternative.

Electricity generated from decentralised smaller utilities is far more efficient, especially when the waste‐heat is being re‐used. Cogeneration facilities, which purposely produce both electricity and heat, are already used in some instances and can recover approximately 80 per cent of the input energy by using the heat that results from electricity production (Hageman, 2020). This has been a known process for centuries, but needs further technological enhancements and political support in order to become a popular means of electricity production again.

In a 2015 World Energy Outlook Special Report by the International Energy Agency (IEA 2015) this view was emphasised: ‘to accommodate high shares of variable renewables, the power system will have to be equipped with the flexibility needed to maintain the reliability of the electricity supply’. An alternative approach to electricity generation that is equipped to meet future demands and developments is available, but underdeveloped in developed economies: distributed energy (or decentralised energy).

Policy making in the energy sector should take this into account and make an informed choice for a more efficient, more reliable, cleaner and economically efficient future of electricity. The EU should speed ahead with a model that focuses fiscal‐stimulus packages on empowering member‐states to support the established of new decentralised renewable energy smart grids, the rapid electrification of transport and mobility infrastructure, integrated with local information‐sharing modalities to enable to rapid sharing and selling of clean energy. This will empower a shift toward localisation and decentralisation of renewable energy production, and provide citizens with greater information control over the uses and distribution of their own energy footprints.

This vibrant new ‘Third Industrial’ model will also be eminently exportable to countries like Malaysia and Brazil, which will be ripe for the introduction of innovative new technology mixes which could transform their societies. Even if the Third Industrial model remains nascent in many EU nations, emerging markets in Asia and Latin American could well provide fertile ground for rapid prototyping and innovation, creating new approaches that could then be applied back in the EU.

## Conclusions

Rather than seeing the Green Deal and Third Industrial Revolution as EU‐centric models, by shifting to a wider global vision, the EU can begin to recognise its opportunity to become the epicentre and export‐driver of a new model of sustainability that will drive the future of global industrial productivity for years to come. In the age of COVID‐19, the need to identify opportunities for economic advancement which respect to planetary boundaries while protecting wildlife and national habitats is essential.

As this report has shown, there is therefore a need to have a coordinated and comprehensive response to COVID‐19 crisis which balances environmental, health and economic concerns.

Politicians and commentators, from across the political spectrum – not to mention many voters – are questioning whether current and conventional economic policies are sufficient to address the challenges and problems their countries face. Many of the policies which have been implemented across Europe not just over the last decade but over the last forty years or so, appear no longer able to improve economic and social outcomes in the ways they once promised.

A lurch into extreme protectionism, which was already undermining the global economy prior to COVID‐19 and now endangers countries securing critical healthcare items, is clearly not the answer. Instead, there is a viable alternative premised on identifying new avenues of cooperation with key emerging markets.

We have shown how strong EU–Asian–American partnerships in response to COVID‐19 can enable all sides to come out stronger and better equipped to tackle future environmental, health and economic crises.

But this requires, in effect, the remaking and redesigning of markets so that while remaining as free and open as possible, they do so now with a clear purpose. Important efforts by national governments in Southeast Asia and Latin America have been highlighted for their potential in establishing the enforceable regulatory regimes that could lay the foundation for genuinely sustainable production processes. Equally, such national regulatory schemes require a combination of robust international scientific oversight mechanisms, as well as far more stronger international financial support. This in turn would open up new EU–Asia–America investment, trade and partnership relationships, potentially seeing the emergence of an international clean ecosystem for the emergence of a joint ‘Third Industrial Revolution’ that could well inspire further transformative changes across other economic regions, if not the world.
